# Long-term outcomes after caudal zona incerta-forel field ablation: three-year clinical follow-up in advanced Parkinson’s disease

**DOI:** 10.3389/fnagi.2026.1756591

**Published:** 2026-03-27

**Authors:** Boris Zurita-Cueva, Luis Vaca-Burbano, Cleto Ramírez-Penso, Mauricio Navarrete-Carreño, Andris Mejía, Juan-Carlos Coronado, Breiner Morales-Asencio, Carol Saldías, Norman López

**Affiliations:** 1 Departament of Neurosurgery, Omni Hospital Guayaquil, Guayaquil, Ecuador; 2 Hospital de la Policía Nacional, Guayaquil, Ecuador; 3 Servicio de Neurocirugía, Centro Cardio-Neuro-Oftalmológico y Trasplante (CECANOT), Santo Domingo, Dominican Republic; 4 Sociedad Dominicana de Neurología y Neurocirugía (SDNN), Santo Domingo, Dominican Republic; 5 Neuroloop Medical Center, Guayaquil, Ecuador; 6 Universidad Central del Este, San Pedro de Macorís, Dominican Republic; 7 Departamento de Procesos Terapéuticos, Facultad de Ciencias de la Salud, Universidad Católica de Temuco, Temuco, Chile; 8 Consorcio Latinoamericano de Investigación (Clati), Temuco, Chile; 9 Escuela de Kinesiología, Facultad de Salud, Universidad Santo Tomas, Temuco, Chile; 10 Departamento de Ciencias Sociales, Universidad de la Costa, Barranquilla, Colombia; 11 Centro de Investigación en Prevención y Cuidados de la Salud (+SALUD), Facultad de Salud, Universidad Santo Tomás, Santiago, Chile

**Keywords:** caudal zona incerta, Fields of Forel, functional surgery, Parkinson, stereotactic ablation, UPDRS

## Abstract

**Introduction:**

Advanced Parkinson’s disease (PD) with a tremor–rigid phenotype poses therapeutic challenges when pharmacological treatments lose efficacy and deep brain stimulation (DBS) is not an option; particularly in contexts where access to implantable neuromodulation therapies is limited. In this scenario, stereotactic ablation of the caudal zona incerta (cZI) and the Fields of Forel has emerged as a surgical alternative, although long-term evidence remains scarce, especially for combined infrathalamic approaches.

**Methods:**

A prospective study was conducted in 10 patients with advanced unilateral PD and a tremor-rigid phenotype, treated with stereotactic radiofrequency in the cZI and Fields of Forel. Motor function was assessed using the UPDRS III at six time points (baseline, 3, 6, 12, 24, and 36 months), allowing the analysis of both early response and long-term clinical stability. Paired Student’s *t*-tests were applied to compare longitudinal changes in UPDRS III between baseline and follow-up assessments. In addition, effect sizes were calculated using Cohen’s d.

**Results:**

UPDRS III scores were significantly reduced at all follow-ups (*p* < 0.001), with mean decreases of −25.8 points at 3 months and −36.3 points at 36 months. Effect sizes were very large (d > 4.0). Tremor improved early and was sustained; rigidity and bradykinesia showed marked reductions during the first year with a slight subsequent upward trend; gait demonstrated continuous progression up to 36 months. No major complications were recorded, and transient adverse effects were minimal, consisting of mild and transient postoperative imbalance in two patients, with rapid clinical recovery and no permanent sequelae.

**Conclusion:**

Stereotactic ablation of the cZI and Fields of Forel provides robust and sustained clinical benefits at 3 years, with a favourable safety profile. These findings highlight the durability of the motor effect of infrathalamic ablative approaches and position this technique as a viable alternative to DBS in selected patients with advanced PD; underscoring the need for multicentre studies to consolidate its role in contemporary functional surgery.

## 1 Introduction

Parkinson’s disease is a progressive neurodegenerative disorder that causes a combination of motor and non-motor symptoms, impairing functional autonomy and quality of life (Abdi-Sargezeh et al., 2024; Ahmed et al., 2023; Aleid et al., 2023). Its prevalence has steadily increased, affecting over 11 million people in 2021, with a disproportionate burden in low- and middle-income countries, where clinical progression tends to have a greater functional impact (Alvarez, 2005; Bajaj et al., 2024). This growth is associated with a higher burden of disability-adjusted life years, especially in middle- and low-income countries (Boogers and Fasano, 2025; Campins-Romeu et al., 2024). In contrast, tremor-dominant or mixed tremor–rigid phenotypes tend to show more heterogeneous clinical courses, with variable responses to dopaminergic treatment and the possibility of developing complex motor symptoms in advanced stages (Chang et al., 2021; Cury et al., 2017; Dahmani et al., 2023).

Deep brain stimulation has been the surgical intervention of choice in patients with advanced disease due to its ability to improve tremor, rigidity, and bradykinesia (Abdi-Sargezeh et al., 2024; Duarte-Zambrano et al., 2025). However, its application faces structural and clinical limitations that reduce accessibility. These include the need for permanent implants, frequent follow-ups, high costs, hardware failures, and anatomical challenges in reaching certain targets (Aleid et al., 2023; Fine et al., 2000). These barriers have driven the development of minimally invasive stereotactic ablative alternatives, which aim to modulate deep circuits through focal lesions, providing comparable motor benefits without implantable devices or prolonged maintenance (Gallay et al., 2020; Godinho et al., 2019; Guridi and Gonzalez-Quarante, 2021).

Historically, ablative techniques have targeted classical structures such as the internal globus pallidus, the subthalamic nucleus, and the ventral intermediate nucleus of the thalamus. These targets have shown sustained efficacy in controlling tremor and other cardinal symptoms (Herrera-Pino et al., 2024; Horisawa et al., 2021; Ikezawa et al., 2025; Kann et al., 2020; Katunina et al., 2025; Kostiuk, 2023). In recent years, less traditional infrathalamic targets such as the caudal zona incerta and the Fields of Forel have been incorporated. Stimulation of the H1 Field has demonstrated clinically significant reductions in MDS-UPDRS III scores, improvement in freezing of gait, and reductions in levodopa dosage over extended follow-up periods (Krishna et al., 2023; Liang et al., 2025; Lozano et al., 1995). In addition, ablative series targeting the VIM and other structures have reported sustained tremor relief in patients with pharmacological refractoriness (Herrera-Pino et al., 2024; Ikezawa et al., 2025; Luo et al., 2025).

In this context, interest has re-emerged in less conventional infrathalamic targets, particularly those that act as strategic anatomical nodes within motor circuits, including the caudal zona incerta and the Fields of Forel. In particular, the Fields of Forel (subthalamic tegmental fields, including H1 and H2) constitute a strategic point of convergence where the main pallidothalamic pathways converge, such as the lenticular fasciculus and the ansa lenticularis, which makes this region a relevant target for interrupting the pathological output of the basal ganglia towards the thalamo-cortical motor circuits (Magnin, 2001; Martínez Fernández et al., 2024; Martínez-Fernández et al., 2020). From this anatomical and pathophysiological perspective, lesional approaches in the field of Forel historically described as campotomy or forelotomy and, more recently, conceptualised as interventions on pallidothalamic pathways have been explored internationally as a functional surgical strategy for the treatment of Parkinson’s motor symptoms, although with less dissemination than classical pallidotomy or thalamotomy (Godinho et al., 2019; Guridi and Gonzalez-Quarante, 2021; Magnin, 2001). infrathalamic structures reinforce the relevance of these structures as convergent nodes for the modulation of motor circuits involved in tremor, rigidity, and other motor symptoms in selected patients with Parkinson’s disease (Gallay et al., 2020; Martínez-Fernández et al., 2023).

Despite these advances, the available evidence presents significant gaps. Most studies on stereotactic ablation including subthalamotomy and focused ultrasound pallidotomy report improvements approaching 50% in MDS-UPDRS III scores at 12-month follow-up, with partial maintenance up to 24 months. However, evidence beyond that period remains limited and heterogeneous, both in motor outcomes and in the stability of benefit (Gallay et al., 2020; Martínez-Fernández et al., 2025a). Moreover, few series have analyzed combined ablation in two infrathalamic targets, and almost none report follow-up windows of 36 months or more (Martínez-Fernández et al., 2025b). Significant gaps also persist in Latin America, where data availability is particularly scarce, and socioeconomic conditions limit access to deep brain stimulation (Campins-Romeu et al., 2024; Navarro-Olvera et al., 2023).

This landscape reveals a clear gap. There is a lack of robust longitudinal evidence evaluating the efficacy and safety of stereotactic ablation targeting the caudal zona incerta and the Fields of Forel, particularly in cohorts with a tremor-rigid phenotype and follow-up periods of 36 months or more. The long-term stability of motor benefits and the potential of these targets as viable alternatives in settings where deep brain stimulation is inaccessible or not indicated also remain unknown (Martínez-Fernández et al., 2025b; Ortega-Robles and Arias-Carrión, 2025; Pal, 2000; Paschen et al., 2025).

Although numerous surgical studies in Parkinson’s disease report results primarily based on short- to medium-term follow-up, generally between 3 and 12 months, these intervals may be insufficient to capture the durability of clinical effects, the emergence of late adverse events, or symptomatic recurrence, aspects that are particularly relevant in ablative procedures with permanent effects (Peng et al., 2025; Peters et al., 2025; Rajabian et al., 2023; Ricciuti et al., 2025). Prolonged follow-up acquires central methodological value, as it allows the evaluation of clinical trajectories beyond the early postoperative effect and the differentiation of sustained benefits from transient improvements, as has been demonstrated in the pallidotomy literature with follow-ups of several years (Rodrigues et al., 2025; Rodriguez-Oroz et al., 2025). Likewise, long-term evidence is increasingly recognised as essential in functional neurosurgery for Parkinson’s disease, including studies with follow-ups close to 36 months, as they more faithfully reflect the true progression of the disease and the sustained clinical relevance of interventions (Sandoval-Pistorius et al., 2023; Shi et al., 2024). In this context, despite the limited sample size, a three-year follow-up provides complementary evidence by directly addressing the durability of the therapeutic effect and long-term safety, aspects that cannot be adequately resolved in studies with short follow-up, even when they include a larger number of patients (Rodrigues et al., 2025; Rodriguez-Oroz et al., 2025; Sandoval-Pistorius et al., 2023).

Therefore, we aimed to evaluate the efficacy and safety of combined stereotactic ablation in the caudal zona incerta and Fields of Forel in a cohort of patients with advanced Parkinson’s disease and a tremor-rigid phenotype. Longitudinal motor changes were assessed using standardized UPDRS III measurements, and clinical stability was analyzed over a 36-month follow-up period, with the goal of contributing evidence to help define the role of these targets in contemporary functional surgery.

## 2 Materials and method

### 2.1 Study design

A longitudinal, prospective observational study was conducted, with a clinical follow-up of 36 months. From a total of 18 participants, 10 adult patients (6 men and 4 women) diagnosed with idiopathic Parkinson’s disease, tremor–rigid phenotype, with predominantly unilateral symptoms, were included. Selection was carried out by a multidisciplinary team comprising specialists in neurology, functional neurosurgery, and neuropsychology.

Inclusion criteria comprised a confirmed diagnosis of Parkinson’s disease according to MDS criteria, refractoriness to dopaminergic treatment, persistent unilateral involvement, and an adequate cognitive and emotional status to comply with the surgical and follow-up process.

The assessment of patients’ cognitive, emotional, and psychiatric status was conducted within the context of the preoperative process through clinical evaluation by an interdisciplinary medical team with experience in the management of Parkinson’s disease and functional surgery. Patients had several years of prior clinical follow-up at the treating centres, which allowed the integration of relevant longitudinal information. The aim of this evaluation, carried out within the framework of clinical meetings and medical boards, was to validate patients’ suitability as candidates for the surgical procedure and to rule out conditions that could contraindicate the intervention or compromise follow-up. The specific instruments and results of these evaluations were not included in the present study. In accordance with this selection process, subjects with severe psychiatric comorbidities, dementia, contraindications to stereotactic surgery, or difficulties adhering to the evaluation and clinical follow-up protocol were excluded.

### 2.2 Pre- and postoperative medical assessments

**Figure 1:**
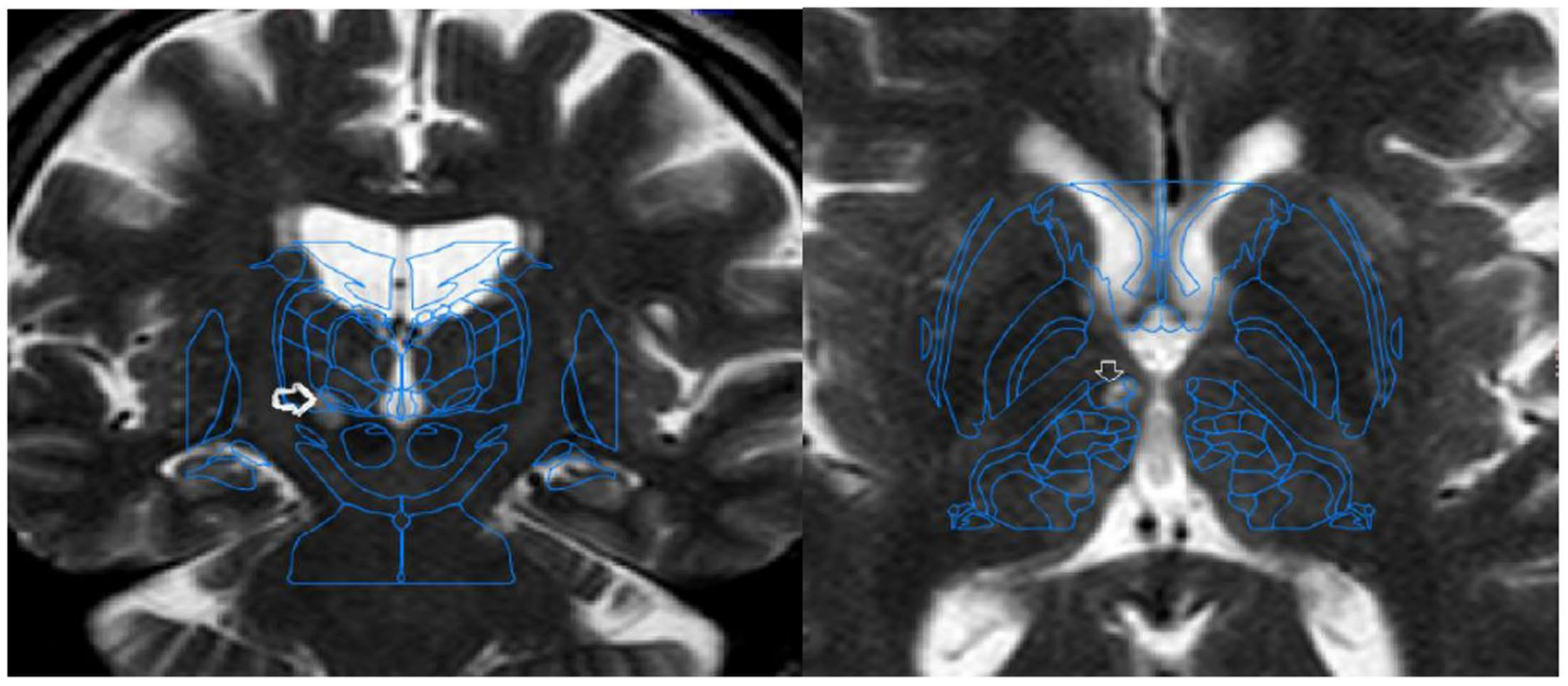
Postoperative coronal and axial T2-weighted MRI images with Schaltenbrand–Wahren atlas fusion, showing microlesions in the caudal zona incerta (left) and fields of Forel (right).

Motor clinical evolution was assessed using the Unified Parkinson’s Disease Rating Scale (UPDRS) Part III, administered by a trained evaluator. The scale was applied at five time points: before surgery (baseline) and during postoperative follow-ups at 3, 6, 12, 24, and 36 months (Figure 1).

### 2.3 Radiofrequency procedure

The intervention was performed using stereotactic techniques with the Blue Frame NS system (FIME, Córdoba, Argentina), together with the MNPS planning system (Mevis, São Paulo, Brazil). The stereotactic radiofrequency ablation procedure was performed with the patient awake, under local anaesthesia, without the use of general anaesthesia. During the intervention, intraoperative clinical monitoring was maintained, which allowed the evaluation of motor response and the detection of potential adverse effects in real time.

The surgical target was defined based on axial T2-weighted magnetic resonance imaging, without the assistance of standardised atlases, prioritising the direct identification of anatomical structures. The caudal zona incerta was established as the target, located medially relative to the posterior border of the subthalamic nucleus, at the level of the plane of greatest diameter of the red nucleus. This region is typically situated 3.5–4 mm below the AC–PC plane.

For precise localisation, image fusion was performed between magnetic resonance imaging and computed tomography obtained with the stereotactic frame. The initial procedure consisted of a thermal intervention targeting the caudal zona incerta using a 2-mm electrode with a 1-mm active tip (Cosman RFG-4A, Burlington, Massachusetts), applying two to three lesions at 75 °C for 60 s, with 1-mm cranial displacements between applications.

Subsequently, the intervention was completed with a pallido-thalamic tractotomy at the level of the Fields of Forel. This was targeted approximately 2 mm posterior to the mid-commissural point, 2 mm below the AC–PC plane, and laterally 7–8 mm from the border of the third ventricle, avoiding the mammillothalamic tract as a critical anatomical reference.

Patients were discharged within the first 24 h after the procedure. Imaging follow-up with magnetic resonance imaging was performed between the first and third postoperative months, with the acquired slices superimposed onto the Schaltenbrand and Morel stereotactic atlas to verify lesion localisation.

Pharmacological management of the patients was carried out under the supervision of the treating neurological team during the preoperative, postoperative, and follow-up periods. As the intervention was indicated in the context of motor symptoms refractory to dopaminergic treatment, no specific protocol for pharmacological adjustment associated with the surgical procedure was established. When necessary, medication modifications were performed on an individualised basis according to clinical judgement and were not included in the analysis of the present study.

### 2.4 Data analysis

A paired-sample Student’s t-test was used to compare mean scores (pre- vs. post-intervention) at each follow-up point. To estimate the magnitude of clinical change, effect sizes were calculated using Cohen’s d, with the following thresholds: small (d = 0.2), medium (d = 0.5), and large (d ≥ 0.8). The level of statistical significance was set at *p* < 0.05. Analysis was performed using IBM SPSS Statistics version 29.0.

### 2.5 Ethical considerations

The study was conducted in compliance with all national and international ethical standards for research involving human participants. Prior to participation, all patients received comprehensive information about the study and signed written informed consent. The research is part of the international project “Analysis of the Effectiveness of Neuromodulation Techniques in Complex and Intractable Medical Conditions” (Code INV.140–04–002-18) and has been approved by the Ethics Committee of the University of La Costa (Approval Code 172/2024), the institution responsible for the study. Patients consented to undergo the indicated procedure and authorized the use of their clinical data for research purposes. All interventions were performed in accordance with the principles of the Declaration of Helsinki and current international guidelines. Procedures were completed without relevant complications and yielded favourable clinical outcomes.

## 3 Results

The cohort consisted of ten patients with advanced Parkinson’s disease, with a mean age of 58.4 ± SD 8.3 years, 7 men and 3 women, with a mean of 11.7 years of education (SD = 2.4), predominantly of South American mestizo background. All patients presented a tremor-rigid phenotype with persistent unilateral involvement and motor symptoms refractory to pharmacological treatment. No systematic record of levodopa equivalent doses or cognitive or non-motor scales was available, as these variables were not part of the objectives of the present study.

Table 1 shows the longitudinal changes in UPDRS III scores during clinical follow-up. Compared to the preoperative stage, a significant improvement in motor performance was observed at all postoperative follow-ups (3, 6, 12, 24, and 36 months), with substantial mean differences and very large effect sizes (Cohen’s d ranging from 2.74 to 4.24).

**Table 1:** Longitudinal changes in UPDRS III scores after surgery.

Comparison		Mean difference	*t*	*p*	Cohen’s d
Pre-surgery	3 meses	22.64	6.14	<0.001	2.74
	6 meses	25.67	7.41	<0.001	3.18
	12 meses	31.08	10.23	<0.001	3.92
	24 meses	32.15	9.15	<0.001	4.24
	36 meses	32.09	9.48	<0.001	4.39
3 months	6 meses	9.7	2.04	<0.001	0.76
	12 meses	7.46	3.16	<0.001	1.32
	24 meses	8.53	3.90	<0.001	2.27
	36 meses	9.17	3.26	<0.001	2.02
6 months	12 meses	5.6	3.06	<0.001	1.39
	24 meses	5.1	1.42	<0.001	1.53
	36 meses	7.22	2.70	<0.001	2.60
12 months	24 meses	−0.78	−0.79	0.79	−0.18
	36 meses	0.92	−0.28	0.38	0.41
24 months	36 meses	1.8	1.49	0.76	0.39

When examining the intermediate follow-ups, the 3- and 6-month evaluations revealed additional significant decreases compared to the assessments at 12, 24, and 36 months. These variations were statistically significant (*p* < 0.001) and showed large to very large effect sizes (d ranging from 0.76 to 2.60), indicating that part of the clinical improvement continued to develop during the first year of follow-up.

Finally, no significant changes in scores were observed between the 12-, 24-, and 36-month follow-ups, suggesting that the greatest reduction in motor symptoms occurs before the first year, while values remain stable from 12 months onward.

**Figure 2:**
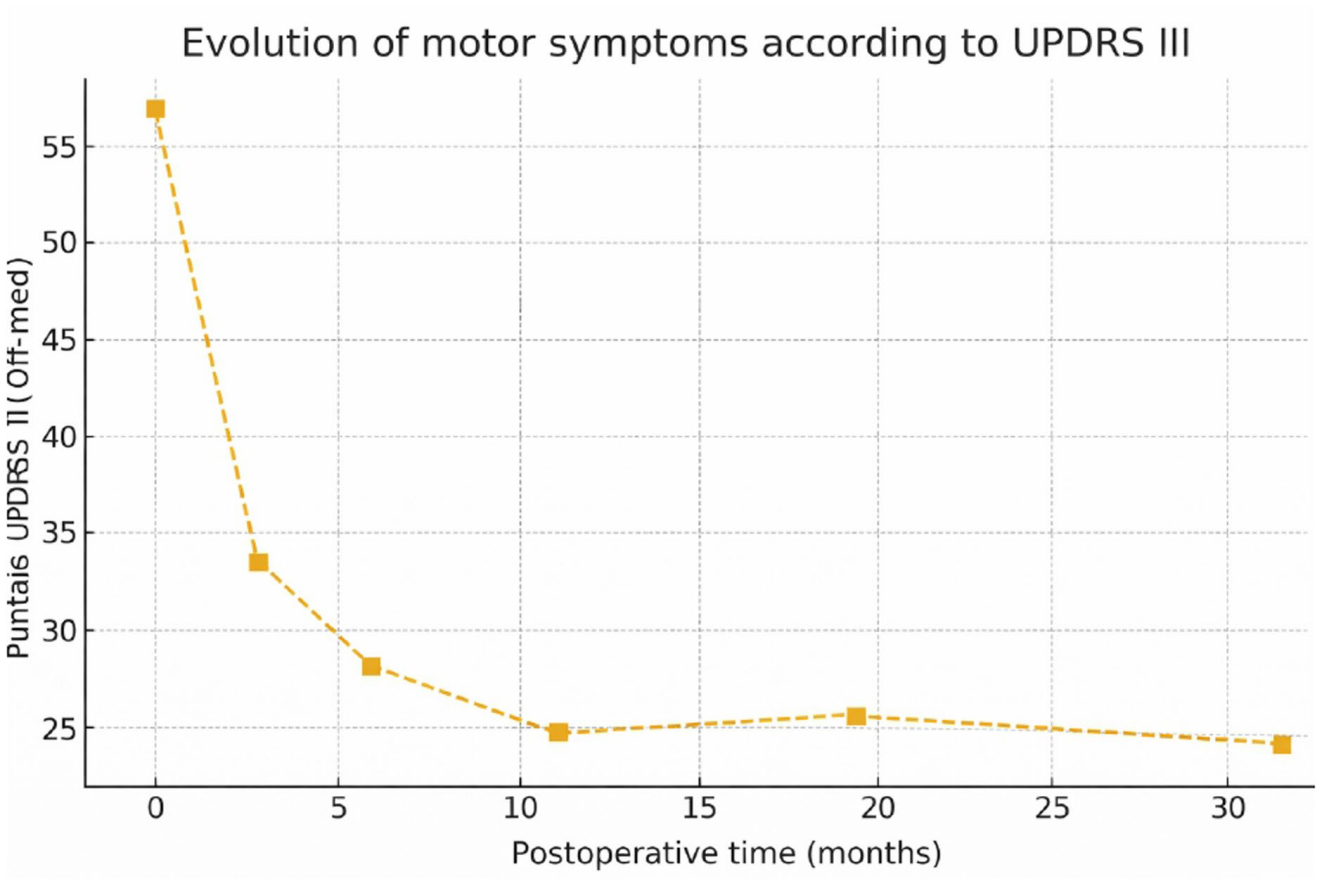
Longitudinal progression of motor scores on the UPDRS III scale (Off-Med).

Figure 2 shows the progression of total motor scores on the UPDRS III scale (in the off-medication condition) in 10 patients with advanced unilateral Parkinson’s disease, evaluated at six time points (pre-surgery and at 3, 6, 12, 24, and 36 months postoperatively). A marked reduction in motor symptoms is observed at 3 months (46.9%), followed by progressive stabilization in subsequent assessments, with sustained improvement maintained up to 36 months. The curve reflects a robust and lasting clinical response to the surgical treatment.

**Figure 3:**
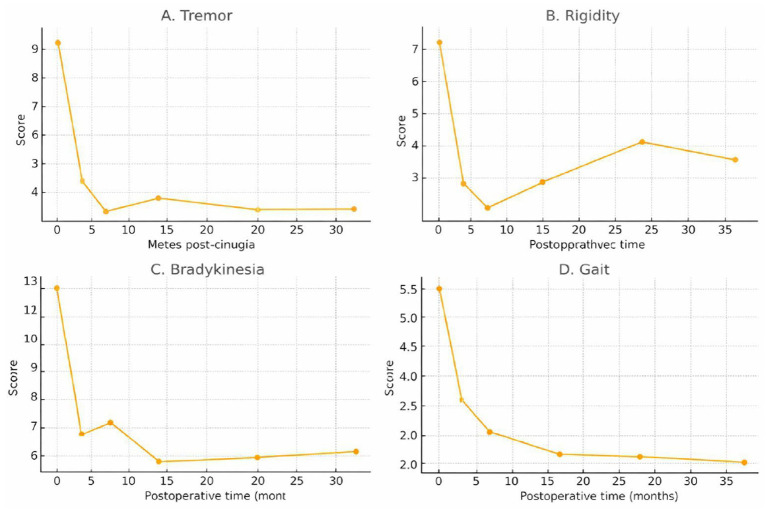
Progression of UPDRS III motor domains in Parkinson’s patients at 36-month post-surgical follow-up: **(A)** Tremor, **(B)** Rigidity, **(C)** Bradykinesia, and **(D)** Gait.

Figure 3 shows the longitudinal evolution of the mean scores in the motor subitems of the UPDRS III: tremor (A), rigidity (B), bradykinesia (C), and gait (D). All evaluated domains showed a substantial reduction during the first 12 postoperative months. Tremor exhibited the greatest early decrease, stabilising at low values throughout follow-up. Rigidity and bradykinesia showed initial improvements followed by slight increases towards 24 and 36 months. The gait domain maintained a sustained downward trajectory, reaching its minimum at 36 months. These patterns reflect a prolonged motor benefit of deep brain stimulation, with a trend towards long-term clinical stabilisation.

In terms of safety, no major surgical complications were recorded during follow-up. Two mild and transient adverse events consisting of postoperative imbalance were observed, which resolved spontaneously during the early postoperative period, without leaving permanent sequelae.

## 4 Discussion

The results from this cohort show that combined stereotactic ablation in the cZI and Fields of Forel provides substantial motor benefits in patients with advanced PD and a tremor-rigid phenotype, particularly during the first year following the intervention. The marked reduction in UPDRS III scores at 3 and 6 months, reflected in very large effect sizes, highlights the ability of these infrathalamic targets to effectively modulate pathological motor circuits. This early response pattern has been previously described in ablative procedures targeting deep brain structures, where focal disruption of hyperactive afferents produces immediate clinical changes (Ikezawa et al., 2025; Sinai et al., 2022).

The stabilization phase between 12 and 36 months is an equally relevant finding. In our cohort, motor scores did not show significant deterioration during this period, suggesting that ablation provides a sustained effect even within a timeframe in which many patients typically exhibit symptom progression. This pattern is consistent with findings from long-term follow-up series of interventions targeting the infrathalamic complex, where clinical benefits have been observed to persist beyond 2 years (Lozano et al., 1995; Souza et al., 2021).

Domain-specific analysis reveals important differences in the dynamics of each motor component. Tremor, for instance, showed the most pronounced early improvement and remained stable throughout the entire follow-up. This outcome aligns with evidence identifying the cZI and the H1 Field as critical nodes in the propagation of abnormal rhythmic oscillations responsible for parkinsonian tremor (Martínez-Fernández et al., 2025b; Souza et al., 2025). The sustained benefit suggests effective and lasting disruption of these circuits.

In contrast, rigidity and bradykinesia showed significant improvements during the first year, followed by slight increases albeit not statistically significant at 24 and 36 months. This pattern is consistent with findings from both ablative therapies and deep brain stimulation targeting other structures, where domains related to slowness and increased muscle tone tend to be more sensitive to neurodegenerative progression (Pal, 2000; Stenmark Persson et al., 2023). These findings suggest that while the initial benefit of ablation is robust, it may be partially modulated by the natural course of the disease.

For their part, gait showed a distinctive behaviour. Unlike the other domains, its improvement continued to progress up to 36 months. This finding is noteworthy, given that axial gait disorders tend to show a lower response to traditional surgical interventions. Studies that have evaluated the stimulation or lesioning of infrathalamic structures have proposed that modulation of these nodes may improve locomotor integration and synchronisation between mesencephalic and thalamic networks (Fine et al., 2000; Stenmark Persson et al., 2022). The positive progression observed in this cohort suggests a possible late reorganisation of these motor networks.

With regard to safety, no major surgical complications were recorded. Two mild and transient adverse events consisting of postoperative imbalance were documented, with rapid recovery and no permanent sequelae, which reinforces the favourable safety profile of the technique. The observed safety can be explained, in part, by precise stereotactic planning and respect for critical anatomical structures in deep regions, a principle widely recognised both in functional neurosurgery and in interventions involving the brainstem and other eloquent areas (Twala, 2025). Recent series of radiofrequency ablation targeting infrathalamic structures have reported similar rates of mild and self-limited events, supporting the reproducibility of these findings (Martínez-Fernández et al., 2025b; Sinai et al., 2022).

In contrast, deep brain stimulation carries risks associated with implanted hardware, including device failure and infections that may require reintervention (Stenmark Persson et al., 2022; Vuletić et al., 2021). Therefore, the ablative option acquires particular relevance in contexts where clinical or resource limitations hinder access to implantable stimulation systems. From a broader clinical perspective, the interpretation of these findings also requires situating them in relation to other functional ablative alternatives currently described in the literature (Magnin, 2001; Waldthaler et al., 2021).

Beyond the comparison with deep brain stimulation, our results should also be interpreted in light of the existing literature on isolated forelotomy and ablation of the caudal zona incerta (cZI). Various international series evaluating interventions on pallidothalamic pathways, including lesions targeting the Fields of Forel, have demonstrated clinically relevant improvements in motor symptoms, particularly tremor and rigidity, with effect magnitudes comparable to those observed in other functional ablative targets (Magnin, 2001; Martínez Fernández et al., 2024; Wang et al., 2025). Similarly, ablation of the caudal zona incerta, especially using modern stereotactic techniques or focused ultrasound, has shown sustained tremor control and motor benefits in selected phenotypes of Parkinson’s disease (Weaver, 2009; Weaver et al., 2012).

In this context, it is relevant to note that the available evidence suggests that isolated forelotomy or ablation of the cZI may generate results of similar magnitude to those observed in the present cohort. In this sense, the potential additional benefit of the evaluated approach should not be interpreted as superiority in terms of early motor improvement, but rather as a broader modulation of interconnected motor circuits at the subthalamic level, which could contribute to greater long-term clinical stability and better adaptation to complex motor phenotypes. This interpretation is consistent with contemporary approaches in functional neurosurgery that prioritise circuit modulation over intervention on a single anatomical target.

Within this framework of comparable efficacy among different ablative targets, follow-up at 36 months constitutes one of the main strengths of this work, as most of the available literature focuses on horizons of 12 to 24 months (Yamamoto et al., 2021). The clinical stability observed during this period provides solid evidence of the durability of the effect at these targets, particularly in Latin American contexts where reports are scarce (Campins-Romeu et al., 2024). This contribution is relevant in a region where access to deep brain stimulation is limited and where viable surgical alternatives are required.

Despite the robustness of the findings, this study has limitations that should be considered when interpreting the results. The reduced sample size and study design limit the ability to establish stronger associations. In addition, the exclusive inclusion of patients with a tremor–rigid phenotype and unilateral involvement restricts generalisation to other clinical profiles. No systematic record of levodopa equivalent daily doses was obtained during follow-up, as pharmacological adjustments were performed according to individual clinical judgement rather than as a structured protocol variable.

Although the complete UPDRS III was applied and specific motor domains were described, the statistical analysis focused on the total score; therefore, it was not possible to draw independent conclusions regarding specific axial symptoms such as freezing of gait. Non-motor symptoms were also not systematically evaluated, as they were not part of the study objectives. Finally, although the 36-month follow-up is one of the most extensive reported for these targets, questions remain regarding stability beyond this period and reproducibility in more diverse populations. These limitations reinforce the need for multicentre studies with larger samples and complementary functional measures to confirm the durability and applicability of these findings.

Taking these considerations into account, it can be concluded that stereotactic ablation targeting the caudal zona incerta and the Fields of Forel is associated with significant, early, and sustained motor improvement in patients with advanced unilateral Parkinson’s disease and a tremor-rigid phenotype. The clinical stability observed over a three-year follow-up, together with the absence of major complications and a favourable safety profile, supports the potential of these infrathalamic targets as a viable therapeutic alternative in selected patients. These findings provide relevant evidence in a regional context with limited availability of deep brain stimulation and reinforce the need for multicentre studies with larger samples that allow a more precise definition of their role within the surgical management of Parkinson’s disease.

## Data Availability

The raw data supporting the conclusions of this article will be made available by the authors, without undue reservation.
